# Increase of retinal layer thickness in a rat model of acute intraocular hypertension

**DOI:** 10.1016/j.exer.2025.110755

**Published:** 2025-11-19

**Authors:** Rui Zhou, Yiwen Li, Rong Wen, Shuliang Jiao

**Affiliations:** a Department of Biomedical Engineering, Florida International University, 10555 W Flagler St, Miami, FL, 33174, USA; b Bascom Palmer Eye Institute, University of Miami Miller School of Medicine, 1638 10 Ave, Miami, FL, 33136, USA

## Abstract

We investigated the retinal response to acute elevation of intraocular pressure (IOP) in rat eyes. IOP elevation was induced in the right eyes of adult Wistar rats using laser photocoagulation of the trabecular meshwork, while the left eye served as untreated controls. Retinal layer thickness was measured *in vivo* using a home-built spectral-domain optical coherence tomography (SD-OCT) system. Glial cell reactivity was assessed using immunofluorescence staining for glial fibrillary acidic protein (GFAP). IOP was significantly increased in treated eyes one day after laser treatment (31.4 ± 6.4 mmHg), returning to normal in most animals by the second day (10.1 ± 2.0 mmHg). We followed the retinal layer thickness for 4 weeks (n = 14) with a subset for 9 weeks (n = 5). OCT images showed a significant increase in retinal layer thickness that persisted for 9 weeks. Histological analysis of retinal sections corroborated the *in vivo* OCT findings. Furthermore, the immuno-biochemical analysis revealed a marked GFAP upregulation, primarily in the retinal nerve fiber layer (RNFL). Quantification of retinal ganglion cells (RGCs) showed no significant loss of RGCs in the treated eyes in ten days after laser photocoagulation. These findings demonstrated that acute IOP elevation in this rat model induces a persisted significant increase in retinal layer thickness, rather than the commonly expected decrease in RNFL thickness. This thickening is associated with the activation of glial cells, suggesting a potential mechanism for this response.

## 1. Introduction

Elevated intraocular pressure (IOP) is a major risk factor for glaucoma, a leading cause of vision loss worldwide (Asrani et al., 2024; Quigley and Broman, 2006). Glaucoma is characterized by the progressive degeneration of retinal ganglion cells (RGCs), their axons within the retinal nerve fiber layer (RNFL) and the optic nerve (Pitha et al., 2024; Vrabec and Levin, 2007). Although the association between high IOP and glaucoma was discovered centuries ago, the mechanisms by which elevated IOP triggers glaucoma are not fully understood. Mechanical stress and vascular compromise due to elevated IOP are implicated (Chan et al., 2017; Feng et al., 2023; Weinreb et al., 2014). Reduction in RNFL thickness is a hallmark of glaucomatous damage (Lommatzsch and van Oterendorp, 2024; Quigley et al., 1980). RNFL thinning is best detected using optical coherence tomography (OCT). OCT has been used for *in vivo* monitoring the status and progression of glaucoma (Lin et al., 2007; Lommatzsch and van Oterendorp, 2024; Schuman et al., 1995).

Animal models mimicking glaucoma induced by elevated IOP provide critical insights into the pathophysiology of the disease (Johnson and Tomarev, 2010; Morrison et al., 2008). In rats, IOP elevation can be achieved by injecting obstructive substances into the anterior chamber to block aqueous humor outflow, by laser photocoagulation of the trabecular meshwork, or by blocking the small veins close to the limbus and episcleral drainage veins (Dey et al., 2018; Sappington et al., 2010; Vidal-Sanz et al., 2012; Zhang et al., 2019). Studies with animal models of glaucoma demonstrated loss of RGCs and thinning of RNFL induced by sustained IOP elevation (Chauhan et al., 2002; Tu et al., 2019).

In the current study, we investigated the effects of acute IOP elevation on retinal layer thickness using spectral-domain OCT (SD-OCT) and histological analysis in a rat model of intraocular hypertension. Contrary to the conventional belief that RNFL thinning is the primary structural change in glaucoma, we observed a sustained increase in retinal layer thickness. Our findings provide new insights into the early retinal response to elevated IOP. This study underscores the importance of reassessing early glaucomatous changes and their potential implications for disease progression and therapeutic intervention.

## 2. Materials and methods

### 2.1. Ethics statement

Experiments involving animals adhered to the ARVO (Association for Research in Vision and Ophthalmology) Statement for the Use of Animals in Ophthalmic and Vision Research and complied with the guidelines established by the Institutional Animal Care and Use Committee (IACUC) of the University of Miami.

### 2.2. Animals, laser photocoagulation, and IOP measurement

Wistar rats (2–3 months old, female) were used. For laser photocoagulation, animals were anesthetized with an intraperitoneal injection of a cocktail containing ketamine (60 mg/kg) and xylazine (8 mg/kg), and topical application of proparacaine 1 % eye drops to the cornea for local anesthesia. A diode laser (NIDEK GYC-1000, NIDEK Inc., San Jose, CA) was used for laser photocoagulation with the following parameters: 532 nm in wavelength, 700 mW in power, 200 μm of spot size, and 0.5 s of duration per spot. The laser beam was focused on the trabecular meshwork, and 40 laser spots were delivered, evenly distributed on the trabecular meshwork of the right eye. The left eye was untreated and served as a control. IOP was measured using a rebound tonometer (TonoLab; Icare Finland Oy, Espoo, Finland) immediately after anesthesia. Both eyes were measured before and daily after laser treatment at approximately 10 a.m. for up to 4 weeks.

### 2.3. In vivo imaging and retinal-thickness calculation

**Fig. 1.**
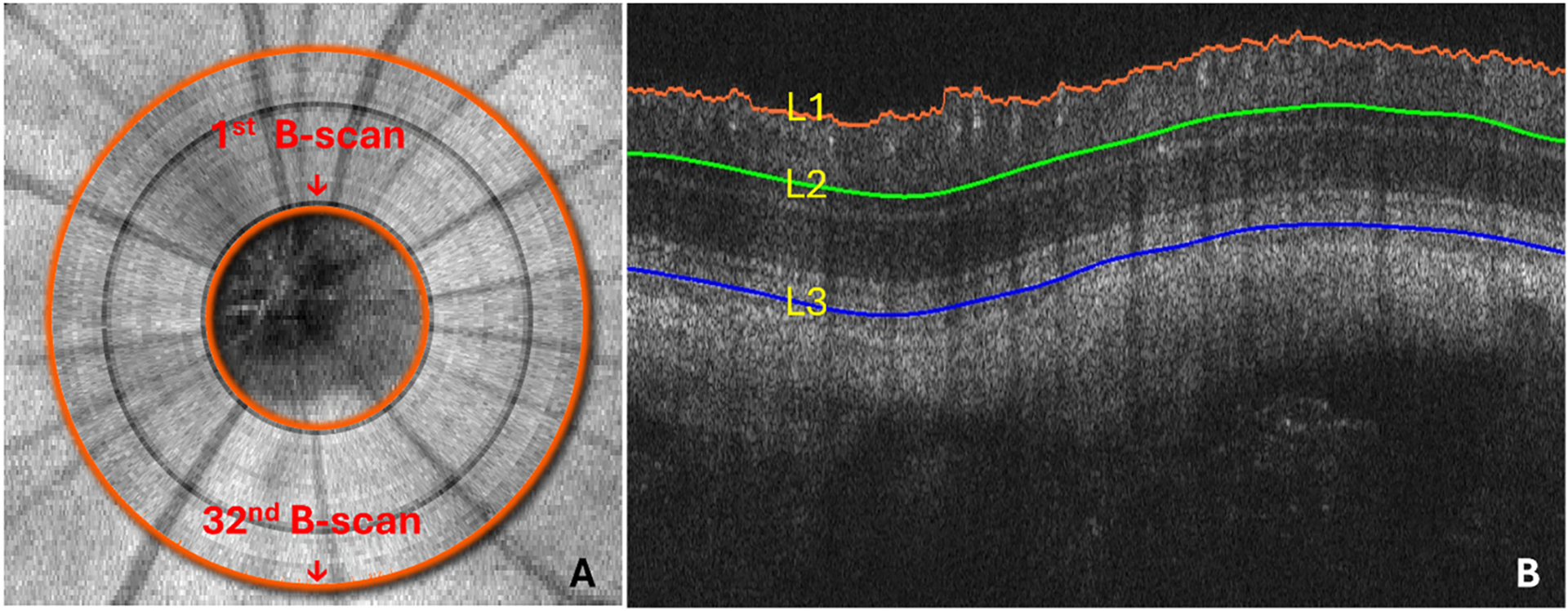
(a) An *en face* view of a 3D OCT data set using the raster scan pattern in which the circular scan pattern was illustrated; (b) a typical circular scan OCT image center at the optic disc with segmentation lines drawn at the ILM (L1), inner edge of INL (L2), and the RPE (L3).

A home-built dual-band SD-OCT was used to image the retina *in vivo*. The system can acquire spatially registered OCT images in the near infrared (NIR, center wavelength: 830 nm) and visible (VIS: center wavelength: 480 nm) simultaneously at an imaging speed of up to 70K A-lines/second. In the current study, we used the NIR-OCT to monitor the thickness of the retinal layers, which has a depth resolution of ~6 μm in air. Both raster and circular scan patterns centered at the optic disc (Fig. 1) were used for imaging. The raster scan consists of 512 (horizontal) × 128 (vertical) depth scans covering an area of 3 mm × 3 mm on the retina. The circular scan has 32 concentric circles with diameters evenly distributed from 1 mm to 2.5 mm, each has 2048 depth scans. Each 3D volume scan took less than 1 s.

We measured the thickness of two regions of the retina, the first is from the inner limiting membrane (ILM) to the inner edge of the inner nuclear layer (INL) containing the RNFL, ganglion cells (GCs), and the inner plexiform layer (IPL). The second contains all the layers of the retina, from the ILM to the RPE. The boundaries of the ILM, INL, and RPE were drawn manually by two operators blinded to the treatment condition of the eyes (Fig. 1). The mean value of the thickness of the retinal layers is calculated as

(1) $$T_{\text{inner}–\text{retina}}=\frac{1}{32\times 2048}\sum_{m=1}^{32}\sum_{n=1}^{2048}\left[Z_{L2}\left(m,n\right)-Z_{L1}\left(m,n\right)\right]$$

(2) $$T_{\text{retina}}=\frac{1}{32\times 2048}\sum_{m=1}^{32}\sum_{n=1}^{2048}\left[Z_{L3}\left(m,n\right)-Z_{L1}\left(m,n\right)\right]$$

where *T_inner-retina_* is the thickness of the inner retina (measured from the inner edge of INL to the ILM). *T_retina_* is the thickness of the full retina from ILM to RPE. *Z*_*L1*_, *Z*_*L2*_, and *Z*_*L3*_ are the depth at locations of L1, L2 and L3 as shown in Fig. 1. The indexes, *m* and *n*, are the number of the circular scans and the number of the depth scans (A-scans), respectively. The standard deviations are calculated accordingly. The thickness of the retinal layers was calculated first in pixels and then converted to micrometers using a calibrated coefficient of 1.29 μm/pixel, which includes the effects of the refractive index of the retina (~1.4).

### 2.4. Histology and immunohistochemistry

For retinal histology, rats were euthanized by CO_2_ overdose 4 weeks after laser photocoagulation, followed by vascular perfusion with mixed aldehydes (LaVail and Battelle, 1975; Song et al., 2003). Eyes were collected and embedded in an Epon/Araldite mixture. Semi-thin sections (1 μm) were cut to display the entire retina along the vertical meridian (LaVail and Battelle, 1975; Song et al., 2003). Retinal sections were stained with toluidine blue and examined by light microscopy.

For glial fibrillary acidic protein (GFAP) immunostaining, animals were euthanized by CO_2_ overdose. Eyes were removed after vascular perfusion with 4 % paraformaldehyde, cryoprotected with 20 % sucrose, and embedded in Tissue-Tek OCT compound (Miles Inc., Elkhart, IN). Sections (10 μm) along the vertical meridian were cut on a Cryostat at −20 °C and thaw-mounted onto Super Frost Plus glass slides (Fisher Scientific, Pittsburgh, PA). Retinal sections were incubated with anti-GFAP antibodies (MA5-12023, Thermo Fisher Scientific Technology, Waltham, MA) at 4 °C overnight. GFAP immunoreactivity was visualized by staining with Cy2 conjugated secondary antibodies (Jackson ImmunoResearch Labs, West Grove, PA) for 1 h at room temperature. Cell nuclei were stained with DAPI (4′,6-diamidino-2-phenylindole). Fluorescent signals in the retinal sections were examined by confocal microscopy (LSM700; Carl Zeiss, Jena, Germany).

### 2.5. RGC quantification

RGCs were identified by immunostaining of RBPMS (Rodriguez et al., 2014), as described previously (Wang et al., 2022). Briefly, eyes were collected ten days after laser treatment. An eyecup was made by removing the anterior segment of each eye and incubated with anti-RBPMS antibodies (GTX118619, GeneTex, Irvine, CA). RBPMS immunoreactivity was visualized with Cy2 conjugated secondary antibodies (Jackson ImmunoResearch Labs, West Grove, PA). After incubating with secondary antibodies, the retina was cut into four quadrants (superior, inferior, nasal, and temporal), flat-mounted onto slides, and examined by confocal microscopy with a field size of 0.581 mm × 0.581 mm. RBPMS positive cells in each quadrant were counted in five fields at 0.5 mm (1 field), 1 mm (2 fields), and 1.5 mm (2 fields) from the optic nerve head. Cell counting was assisted by CellProfiler (V4.2.8, Broad Institute, Inc.). The numbers of RGCs in the 20 images (five fields in four quadrants) from the same retina were averaged and RGC densities were calculated.

### 2.6. F-actin staining and confocal imaging

In a parallel experiment for examining damage to the cytoskeletons of the RNFL we used confocal microscopy to image F-actin. The rat eyes were treated with more laser spots resulting in an elevated IOP sustained for 3 days. The procedure for F-actin staining is similar to that reported in the literature (Huang et al., 2006) with minor modifications. Briefly, eyes were enucleated after vascular perfusion with 4 % paraformaldehyde. A 5-mm diameter eyecup containing the optic nerve was dissected. The tissue was permeabilized in phosphate buffered saline (PBS) containing 0.8 % TritonX-100 for 1 h and then incubated in blocking solution (5 % bovine serum albumin and 0.8 % Triton X-100 in PBS) for 1 h at room temperature. The tissue was thoroughly washed in PBS and then incubated with Alexa Fluor^®^488 conjugated phalloidin (1:40 in PBS, Invitrogen) for 1 h at room temperature to label F-actin. The retina was then rinsed thoroughly, flat-mounted on slides, and stored at 4 °C until imaging. The retinal flat mounts were imaged using a confocal laser scanning microscope (Leica TCS SP5, Leica Microsystems, Bannockburn, IL). Enface images were acquired using 40 × and 63 × oil-immersion objectives with a field size of 387.5 μm × 387.5 μm and a sampling density of 0.76 μm/pixel.

### 2.7. Statistical analysis

Data are presented as mean ± standard deviation (SD). Full retinal thickness and inner retinal thickness measured with OCT between treated and control eyes was compared at each week using paired t-tests performed by R version 4.5.0. RGC densities between laser treated and control eyes were compared and analyzed using paired *t*-test. A p-value of <0.05 was considered statistically significant.

## 3. Results

### 3.1. Laser photocoagulation induces acute IOP elevation

The IOP of each eye was measured the day before laser photocoagulation and each day after treatment until that of the treated eyes returned to baseline. The frequency of measurement was limited to minimize potential corneal damage.

**Fig. 2.**
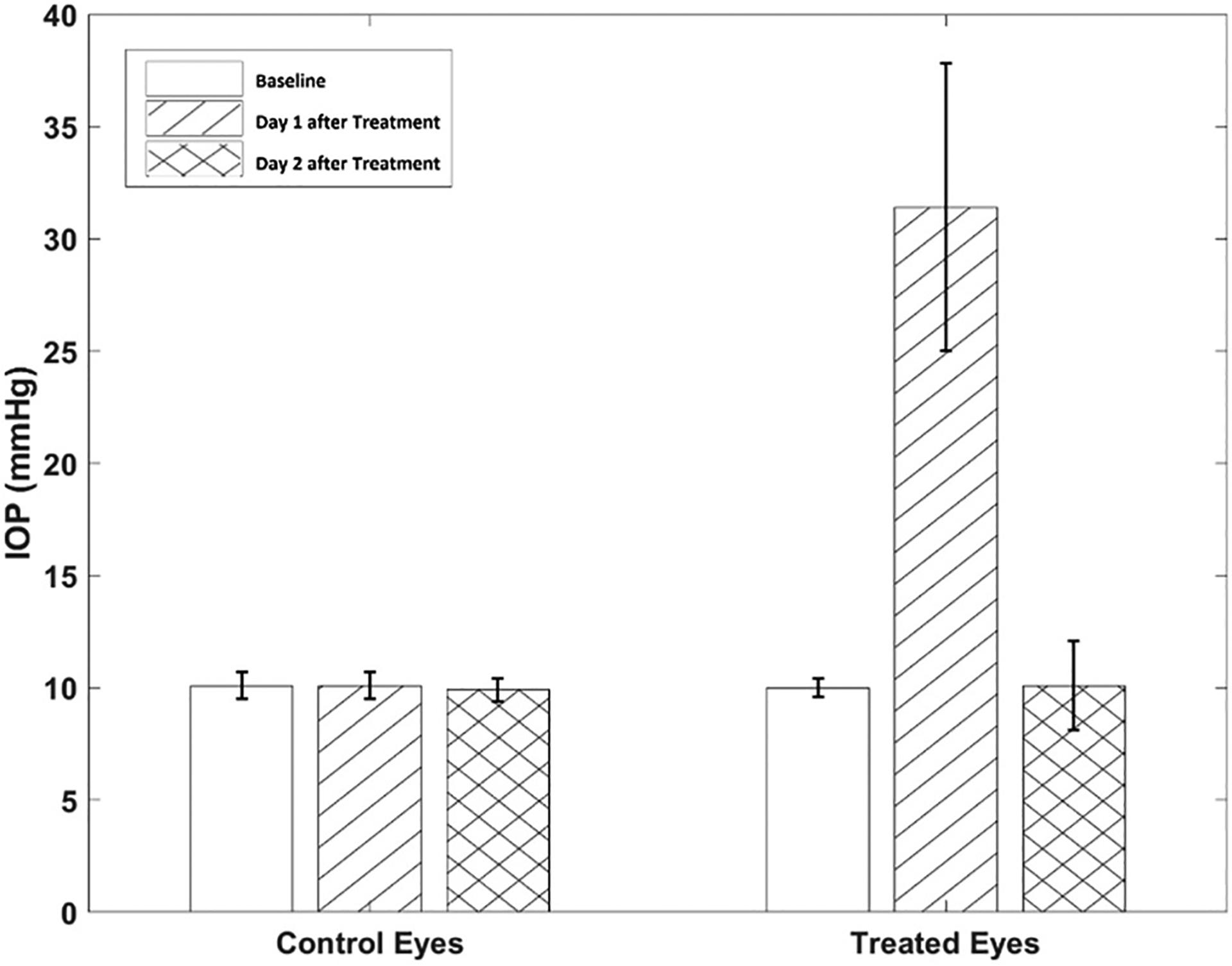
IOP alteration after laser treatment. The IOP (mean ± SD) in the treated eyes were significantly elevated on day 1 and returned to the normal level on day 2 after treatment. No significant changes occurred in the control eyes.

Of the 20 rats initially treated, 14 were followed with retinal imaging for four weeks, in which five were followed for nine weeks. Six were excluded from follow-up due to severe complications or difficulties in obtaining clear retinal images. Fig. 2 shows a significant IOP elevation in the treated eyes of the 14 rats one day after treatment. No significant change in IOP was found in the untreated eyes (Fig. 2). The IOPs in the treated eyes returned to the baseline levels (Fig. 2) two days after the treatment. No noticeable fluctuations in IOP were observed in the 4-week period.

### 3.2. Increase in retinal thickness following laser photocoagulation

**Fig. 3.**
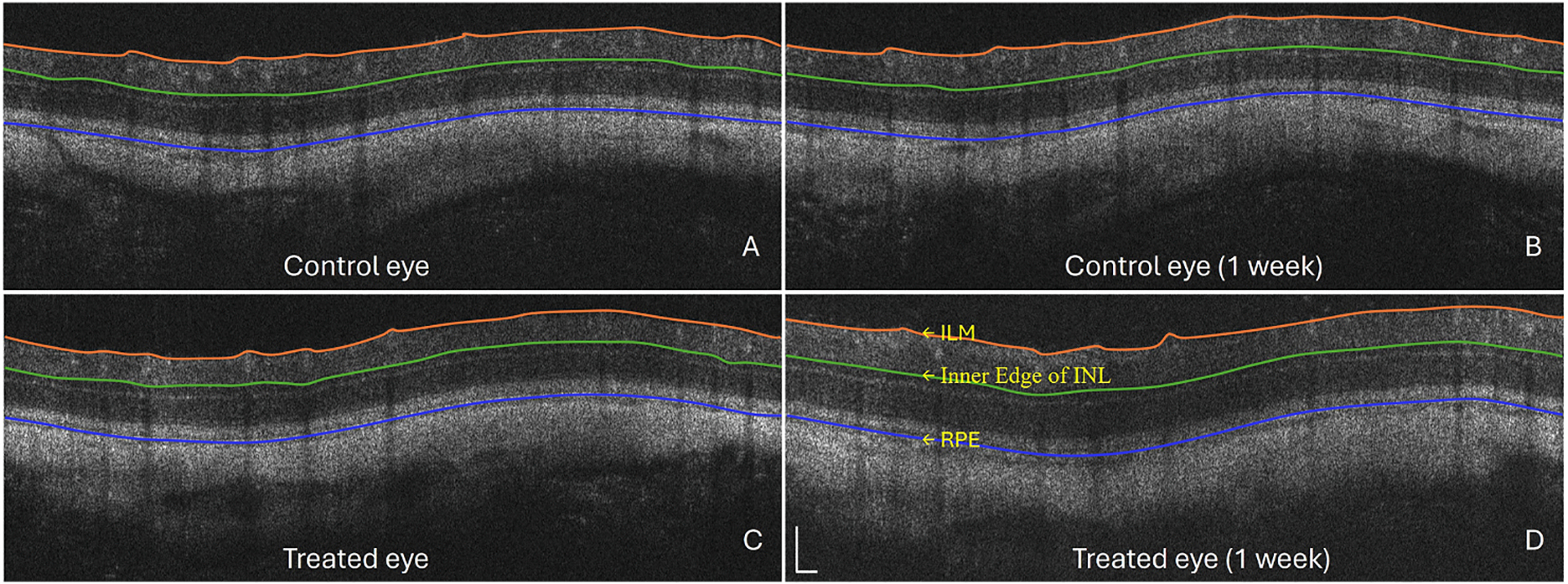
OCT images show the retinal thickness change before and 1 week after laser treatment. In the control eye, no noticeable change in retinal thickness was observed (A, B), whereas in the treated eye, the retina was thickened (C: before treatment; D: 1 week after treatment). All the images are aligned at the RPE layer for comparison. Orange, Green, and blue lines correspond to L1, L2, and L3 in Fig. 1. Scale bars (horizontal and vertical): 100 μm.

We imaged the retinas weekly for 4 weeks post-treatment, in which 5 rats were imaged for 9 weeks using OCT. In many eyes, the corneas were slightly opaque 1 day after laser treatment when the IOP was high. The corneas typically cleared within 2–3 days, allowing for good quality OCT images to be obtained starting one week after laser treatment (Fig. 3).

OCT images revealed that the retina in the treated eye (Fig. 3D) was significantly thicker than at its baseline (Fig. 3C) and compared to the retina in the control eye (Fig. 3A and B). No significant changes in retinal thickness were observed in the control eyes before and after laser treatment.

**Fig. 4.**
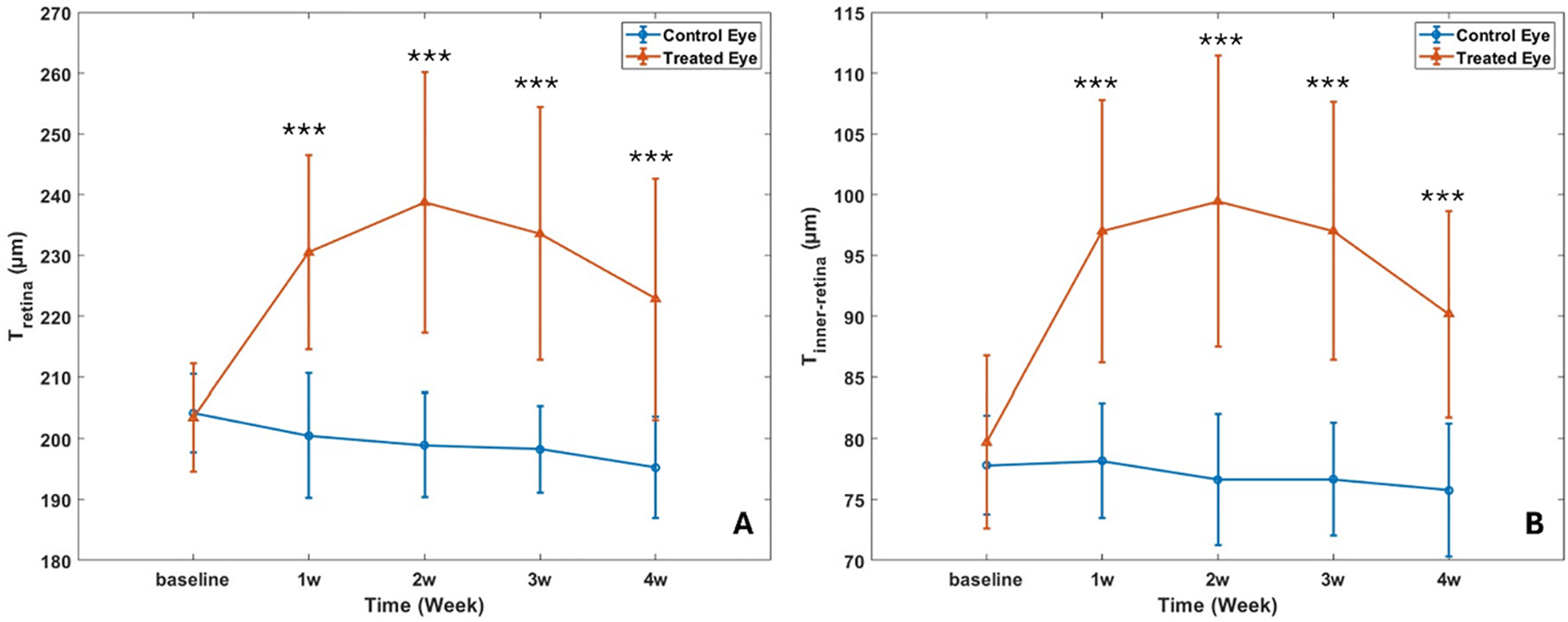
Increase in retinal layer thickness. The full retinal thickness in the treated eye increased significantly one week after laser treatment. The increase peaked in week 2 after laser treatment and lasted through week 4 (A). The increase in the thickness of the inner retina in the treated eyes followed a similar pattern as the full retina thickness (B). No significant changes occurred in the control eyes. Data are presented as Mean ± SD. Asterisks (***) denote the existence of a highly significant difference (p < 0.001) between control and treated eyes at the same week.

The average thickness of the full retina and inner retina were calculated using Eq. (1) and Eq. (2), respectively. The thickness of the full retina in the treated eyes increased significantly one week after laser treatment, as compared to the baseline and the control eyes. The retinal thickness of the treated eyes reached its peak in week 2 and remained significantly thicker for four weeks after laser treatment (Fig. 4A).

The thickness of the inner retina also increased in a similar manner to the full retinal thickness (Fig. 4B). However, percentagewise, the increase in inner retinal thickness was greater than that in the full retina. Data for retinal thickness and inner retinal thickness are summarized in Tables 1 and 2, respectively.

**Fig. 5.**
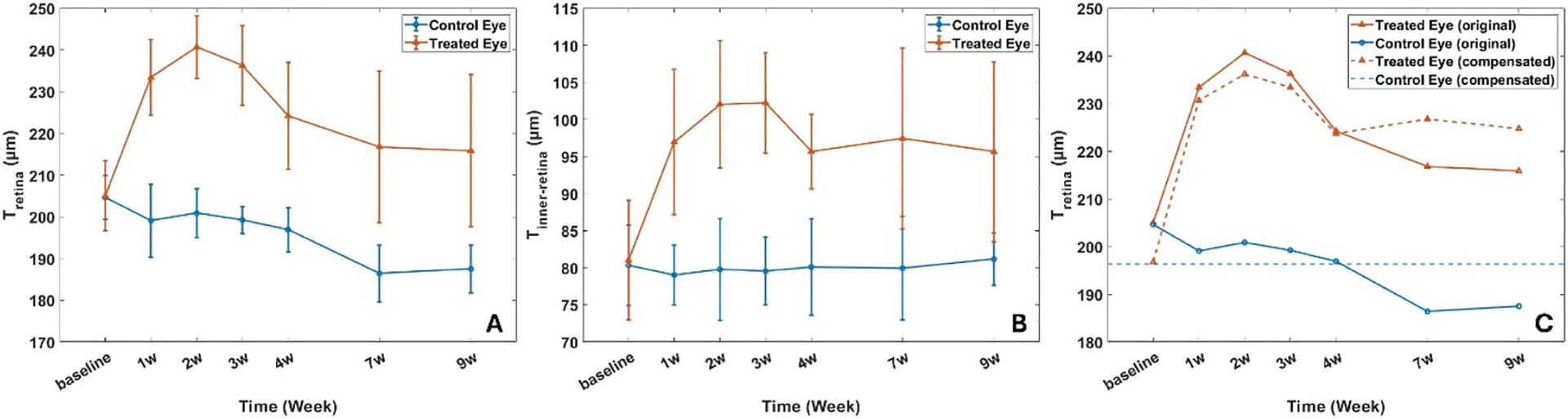
T_retina_, T_inner-retina_, and T_retina-comp_ of a subset (n = 5) of the 14 rats in 9 weeks after laser photocoagulation. T_retina_ (A) and T_inner-retina_ (B) remained increased up to 9 weeks, despite a partial reduction from 2 to 9 weeks. After compensation for potential systematic errors from the imaging process or natural age-related retinal thinning, we can see that T_retina_ and T_inner-retina_ showed similar trends (C).

For the five rats that followed for 9 weeks, the measured T_retina_ and T_inner-retina_ of the control and treated eyes are shown in Fig. 5. T_retina_ and T_inner-retina_ of the treated eyes peaked at around week 2, then declined. In the 9-week period, T_retina_ of the control eyes declined continuously, and the treated eyes had a similar trend (Fig. 5). Focusing adjustment of the ocular lens and polarization mismatch between the sample and reference arms of the OCT system can cause variations of the structural appearance of the retinal layers especially for the RPE, which may affect segmentation of the retinal layers. We can use the apparent decline of T_retina_ in the control eyes to compensate for the effect of the unknown factors: *T*_*retina*–*comp*_ = *T*_*retina*–*treat*_^*−*^ [*T*_*retina–contr*_ − *mean*(*T*_*retina*–*contr*_)], where *T*_*retina*–*comp*_ is the compensated retinal thickness, *T*_*retina–treat*_ is the original calculated retinal thickness of the treated eye, *T*_*retina*–*contr*_ is the calculated retinal thickness of the control eye, and the mean is calculated over the 9 week period. The compensating results are shown in Fig. 5c. Our compensation method may provide a way to isolate the retinal thickening from effects not related to the acute IOP elevation, such as the possible age-related retinal thinning as reported in rats (Nadal-Nicolas et al., 2018).

From the compensated results (Fig. 5c) for T_retina_ shown in Fig. 5a, we can see that T_retina_ and T_inner-retina_ showed similar trends. The thickness of both the full retina and the inner retina declined from the peak at week 2 until week 4, then remained thickened for the rest of the 9 weeks.

### 3.3. Retinal morphological analysis

To confirm the IOP-induced retinal thickness increase measured *in vivo*, we performed retinal morphological analysis. Both control eyes and treated eyes were collected 4 weeks after laser treatment, and semi-thin plastic sections were examined by light microscopy.

**Fig. 6.**
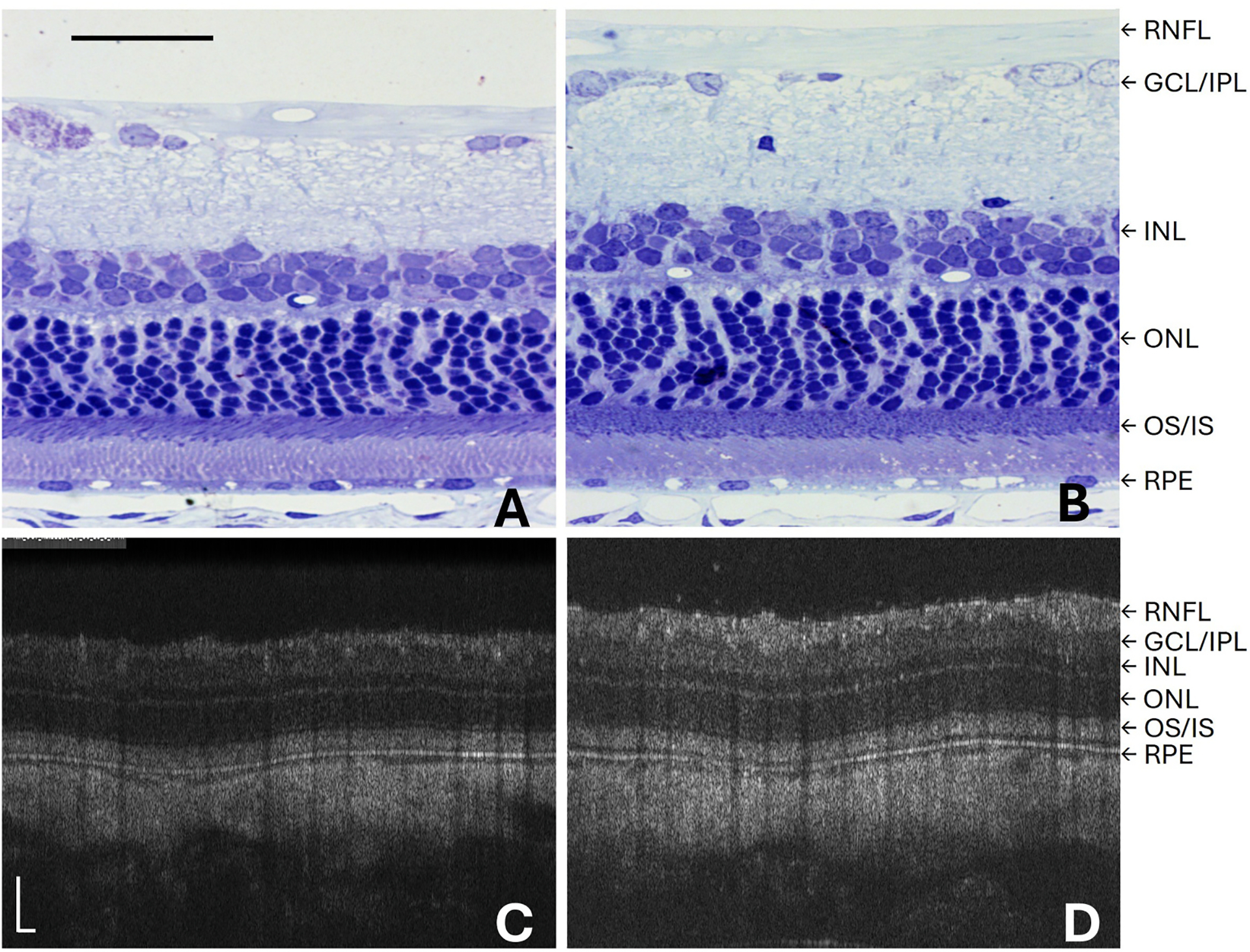
Morphological analysis of the retina. Semi-thin retinal sections from an animal four weeks after laser treatment show that the increase in thickness crosses every layer of the retina in the treated eye (B) compared to the untreated control eye from the same animal (A). The increase is more significant in the inner retina, especially in the RNFL (B), which is almost twice as thick as that in the control eye (A). Panels C and D are the OCT images from the control eye and treated eye, respectively, of the same rat. Scale bars: 30 μm for A and B, 100 μm for C and D (horizontal and vertical).

Representative retinal sections are presented in Fig. 6. The retina in the laser treated eye (Fig. 6B) was noticeably thicker than the untreated eye (Fig. 6A). The increase in retinal thickness extended across the entire retina, especially in the RNFL. The retinal OCT images of the control eyes (Fig. 6C) and the treated eyes (Fig. 6D) of the same rat taken at four weeks after laser treatment are consistent with the histological findings.

### 3.4. GFAP immuno-biochemical analysis

**Fig. 7.**
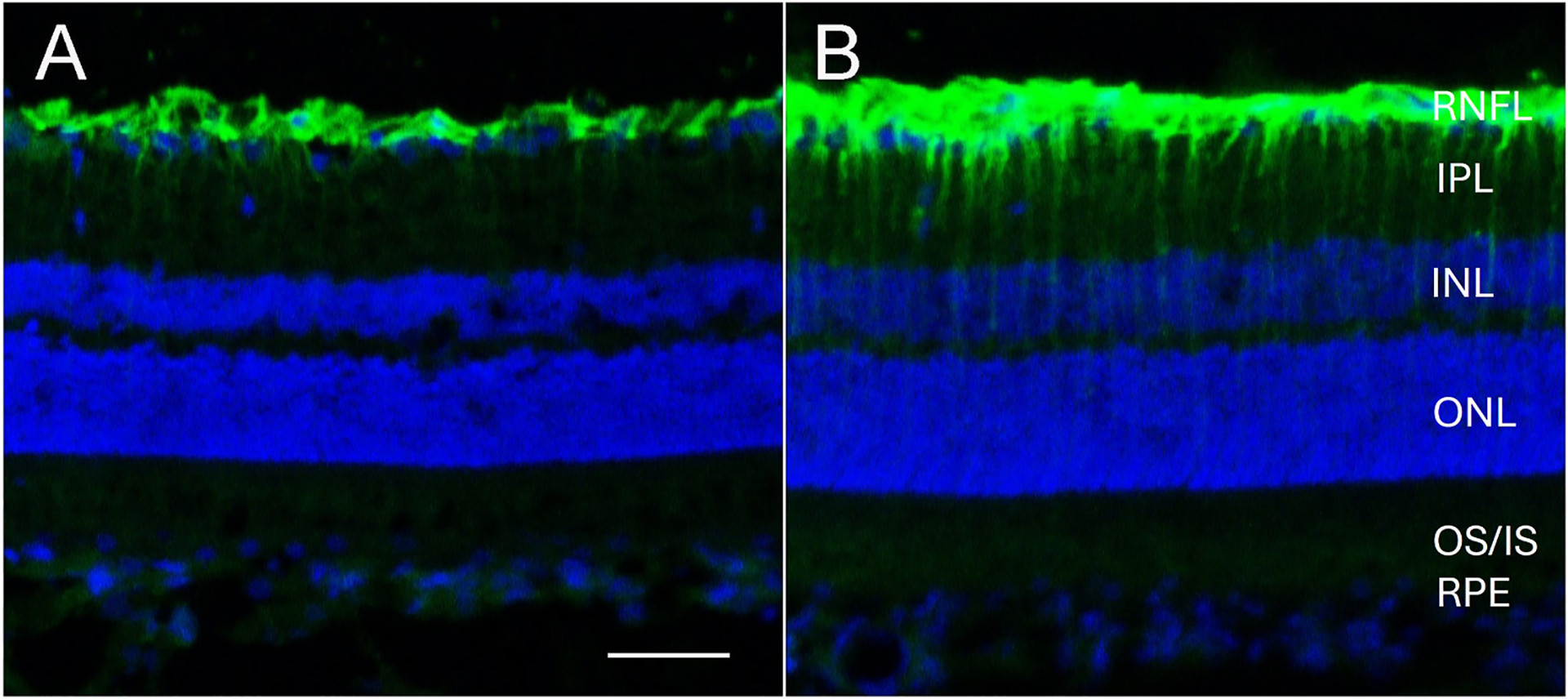
GFAP immune-biochemical analysis. Retinal samples were from a rat four weeks after laser treatment and stained with anti-GFAP antibodies. GFAP staining (green) increased in the treated eye (B), compared to the untreated control eye (A) from the same rat. The increase in GFAP staining was observed mainly in the Müller cell endfeet on the inner border of the retina where RNFL locates (B). Scale bar: 50 μm.

To investigate the activation of glia cells, we examined GFAP expression in the retina using immunocytochemistry. As shown in Fig. 7, GFAP staining is greatly increased in the treated eye (Fig. 7B) as compared to the control eye (Fig. 7A). The increased GFAP staining was predominantly localized in the Müller cell endfeet at the inner surface of the retina overlapping the RNFL, consistent with the noticeable increase in the RNFL thickness shown in the retinal histological section (Fig. 6). The finding that the increase in the retinal thickness extends to whole retina suggests that Müller cells are likely the primary cells involved, since they are the only cell type that span the retina from the ILM to the outer limiting membrane (OLM). Upregulation of GFAP in contralateral eye in a mice model of laser-induced ocular hypertension was reported (Gallego et al., 2012), which cannot be excluded in the current study. However, this possible upregulation of GFAP does not thicken the retina of the control eyes as shown in Figs. 4 and 5.

### 3.5. RGCs quantification and F-actin Organization in the RNFL

**Fig. 8.**
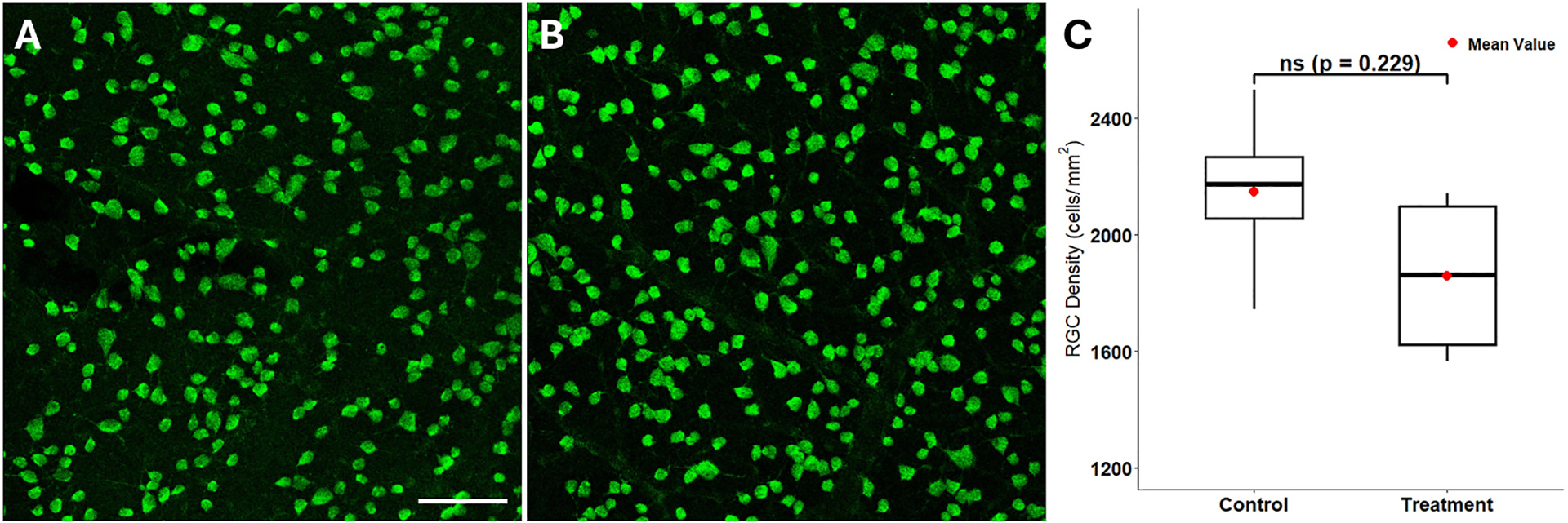
RGC quantification. Representative confocal images of retinal flat mounts show RGCs from a control eye (A) and a treated eye (B). RGCs were identified by RBPMS immunoactivity (green). Quantification analysis is presented as RGC densities (C). No statistically significant difference between the two groups (n = 4) was found (p = 0.229, paired *t*-test). Scale bars: 100 μm.

To investigate potential RGC loss induced by the acute intraocular hypertension, RGCs were quantified 10 days after laser treatment. The mean RGC density was 2148 ± 310 cells/mm^2^ (n = 4) in control eyes and 1859 ± 295 cells/mm^2^ (n = 4) in laser treated eyes. No significant difference in RGC density was found between the two groups (p = 0.229, paired *t*-test), indicating no significant RGC loss had occurred (Fig. 8).

**Fig. 9.**
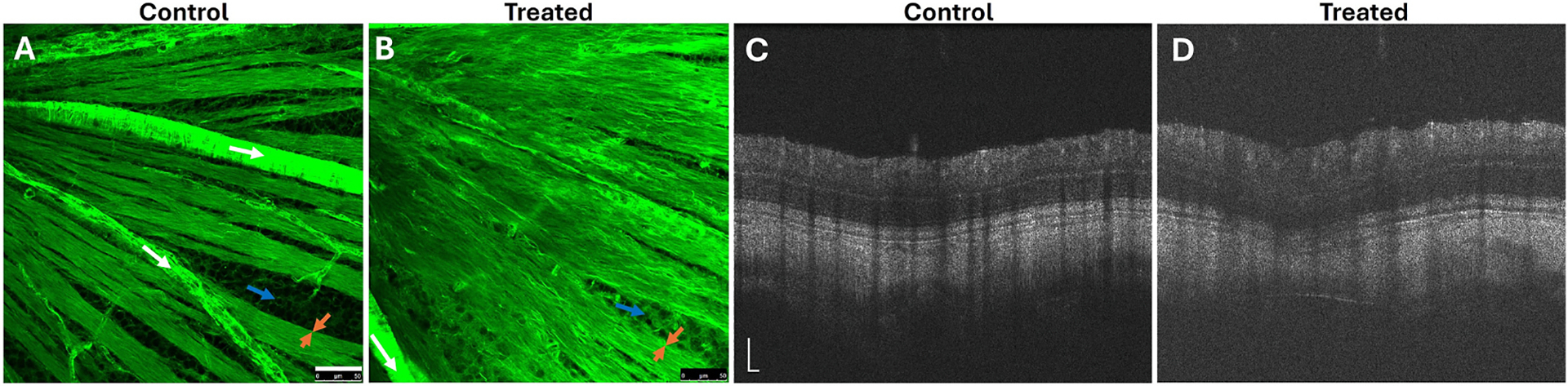
Images of a rat retina in a parallel study with elevated IOP sustained for 3 days. Confocal imaging of retinal flat mounts stained for axonal F-actin for the control (A) and the treated eye (B). Alterations in the F-actin bundles are noticeable in the treated eye. OCT images of the retina for the control (C) and treated eye (D). The retina was thickened in the treated eye. Orange arrows: Boundary of the bundle; Blue arrow: Inter-space between bundles; White arrow: Blood vessel. Scale bars: 50 μm for (A) and (B), 100 μm for (C) and (D).

In the parallel experiment for examining potential damage to the cytoskeletons of the RNFL, a group of ten rats were studied. We observed distortions of F-actin in the RNFL, indicating alterations to the retinal nerve fiber bundles, together with thickening of the retinal layers including the RNFL (Fig. 9). Diffused boundaries and indiscernible inter-space (blue arrow) among bundles can be observed in the treated eye. In addition, a notable difference in thickness between the control eye (Fig. 9c) and the treated eye (Fig. 9d) can be seen in the OCT images from the same rat before confocal imaging. The observation indicates that structural alteration in retinal nerve fiber bundles and retinal layer thickening may happen concurrently.

## 4. Discussion

We have demonstrated a significant increase in the retinal thickness of a rat model in response to acute intraocular hypertension. Our *in vivo* OCT monitoring for 9 weeks showed that the increase in retinal thickness was significant at week 1, peaked at week 2, and maintained throughout the ninth week. The increase in retinal thickness was throughout all retinal layers but is predominantly in the inner retina (measured from the ILM to inner edge of INL) in terms of percentage, especially in the RNFL. The *in vivo* OCT finding was confirmed by *ex vivo* morphological analysis. The retinal thickness increase observed in the present study is likely an early response of the retina to IOP elevation. Compared to the studies in eyes with chronic IOP elevation (Chauhan et al., 2002; Tu et al., 2019), a short-term 1–2 day IOP elevation in the current study makes it possible to isolate early retinal responses before thinning of the RNFL occurs.

The retinal thickening in response to IOP elevation raised a question of what the underlying mechanism(s) is, and which cell types are involved. Since the increase is throughout all the retinal layers, and Müller cells are the only cell type that extend to all layers of the retina, we hypothesized that Müller cells were most likely to be involved. Our immunohistochemistry study revealed that an increase in GFAP staining located in the Müller cell endfeet overlapping the RNFL, supporting our hypothesis that Müller cells may be activated in response to IOP elevation. Müller cells are the principle glial cells that provide structural and functional support to the retina (Sarthy and Ripps, 2001). They express mechanoreceptor TRPV4 and are regarded as the main retinal mechano-sensors (Prieto-Lopez et al., 2024). It is conceivable that Müller cells sense IOP elevation and then are activated to strengthen the architecture of the retina by elongating their cell bodies or perhaps combined remodeling the extracellular matrix (Prieto-Lopez et al., 2024). As part of the RNFL, the thickened Müller cell endfeet may lead to an increase in RNFL thickness. While our results point to the involvement of Müller cells in whole retina thickness change, the contribution of astrocytes for RNFL thickening should also be considered. Astrocytes are almost entirely restricted to RNFL, and therefore, they might not be directly involved in the thickening of the whole retina except the RNFL. Since GFAP labels both reactive Müller cells and astrocytes, and astrocytes are known to become reactive under elevated IOP (Wang et al., 2002), their involvement in the RNFL thickening cannot be excluded.

Thickened RNFL has been reported in a rat model of acute IOP elevation (50 mmHg for 8 h), in which the RNFL thickness increased by up to 15–19 %, and the thickening effect lasted for 3 weeks (Abbott et al., 2014). It also has been shown that 3–12 days transient RNFL thickening can occur as an early inflammatory response in a rat model of optic nerve crush (Rovere et al., 2015). Our model features an acute (1–2 day) IOP spike, which could be particularly relevant to clinical scenarios of acute IOP elevation, such as primary angle-closure glaucoma (PACG) or secondary angle-closure glaucoma (SACG). In contrast to chronic glaucoma models, which mainly aim to replicate the slow, progressive nature of glaucoma, findings from our study highlight a distinct early phase of retinal response that may be masked or absent in models of sustained, slow IOP elevation. In human patients with PACG, an increase in RNFL thickness was reported to last for more than 2 weeks (Liu et al., 2010). While these publications only concentrated on the RNFL, our findings revealed that all the retinal layers are thickened in response to the acute IOP elevation.

The observation of GFAP upregulation may indicate that the optical reflection of the retinal nerve fiber bundles reported in the literature (Huang et al., 2011, 2012) contain the increase of reflection from GFAP in the background. To directly test this hypothesis, future experiments could employ co-staining for GFAP and F-actin in retinal flat-mounts to check contribution of GFAP to the calculated reflection of the retinal nerve fiber bundles.

We found no significant difference in RGC density between control and treated eyes at ten days post-treatment despite observed concurrent structural alterations to the RNFL via F-actin staining and significant thickening in retinal layers. This finding suggests that either the initial retinal thickening and cytoskeletal disruption in the RNFL precedes detectable RGC loss in this model of acute IOP elevation or the thickening of the retina and Muller cell activities induced by the acute intraocular hypertension in our present study could be a protective reaction of the retina. Future studies will seek to clarify whether this thickening phase represents a potentially reversible, neuroprotective state or is an early sign of an irreversible degenerative cascade. Finally, this study was conducted exclusively in female rats; therefore, these findings may not be generalizable to males without further investigation.

## 5. Conclusion

We observed that acute IOP elevation leads to a significant and rapid thickening of all the retinal layers in a rat model of laser photocoagulation. Activated glia cells likely contribute to the thickening of the retina. The present study highlights the importance of considering retina thickening in the early stage of IOP elevation. Investigating the mechanisms underlying the thickening response could enhance our understanding of glaucoma and potentially lead to novel therapeutic approaches.

## Figures and Tables

**Table 1:** Full retinal thickness.

Weeks		Control Eyes (Left)		Treated Eyes (Right)		*P value*
		Thickness (μm)	Change (%)	Thickness (μm)	Change (%)	
Full dataset (n = 14)	0	204 ± 6	–	203 ± 9	–	*0.480*
	1	200 ± 10	−1.9 ± 2.8	230 ± 16	13.4 ± 7.3	<0.001***
	2	199 ± 9	−2.7 ± 1.6	239 ± 22	17.2 ± 8.1	<0.001***
	3	198 ± 7	−3 ± 1.4	234 ± 21	14.8 ± 8.3	<0.001***
	4	195 ± 8	−4.5 ± 2.3	223 ± 20	9.5 ± 7.7	<0.001***
Subset (n = 5)	0	205 ± 5	–	205 ± 9	–	*0.877*
	1	199 ± 9	−2.7 ± 2.4	233 ± 9	14 ± 5.6	*0.004* **
	2	201 ± 6	−1.9 ± 0.7	241 ± 8	17.5 ± 4.2	<0.001***
	3	199 ± 3	−2.6 ± 1.3	236 ± 10	15.4 ± 5.6	<0.001***
	4	197 ± 5	−3.8 ± 1.8	224 ± 13	9.5 ± 5.8	*0.011* *
	7	186 ± 7	−9 ± 2.5	217 ± 18	5.9 ± 8.1	*0.023* *
	9	187 ± 6	−8.4 ± 3.5	216 ± 18	5.3 ± 8.7	*0.043* *

Data are presented as Mean ± SD. The difference between control and laser treated eye was analysis by paired *t*-test.

* p < 0.05.

** p < 0.01.

*** p < 0.001.

**Table 2:** Inner retinal thickness.

Weeks		Control Eyes (Left)		Treated Eyes (Right)		*P-value*
		Thickness (μm)	Change (%)	Thickness (μm)	Change (%)	
Full dataset (n = 14)	0	78 ± 4	–	80 ± 7	–	*0.175*
	1	78 ± 5	−0.5 ± 6.3	97 ± 11	22.2 ± 12.5	<0.001***
	2	77 ± 5	−1.5 ± 2.5	100 ± 12	24.9 ± 10.8	<0.001***
	3	77 ± 5	−1.4 ± 2.6	97 ± 11	22.1 ± 11.6	<0.001***
	4	76 ± 5	−2.9 ± 3.4	90 ± 8	13.5 ± 10.7	<0.001***
Subset (n = 5)	0	80 ± 6	–	81 ± 8	–	*0.826*
	1	79 ± 4	−1.6 ± 3.8	97 ± 10	20 ± 8.5	*0.006* **
	2	80 ± 7	−1.1 ± 2.4	102 ± 9	26.5 ± 6.2	<0.001***
	3	80 ± 4	−0.7 ± 2.2	102 ± 7	27 ± 9.1	<0.001***
	4	80 ± 7	−0.8 ± 3	96 ± 5	18.9 ± 9.5	*0.004* **
	7	80 ± 7	−0.6 ± 2.5	97 ± 12	21.5 ± 20.1	*0.066*
	9	81 ± 3	1.2 ± 3.4	96 ± 12	19 ± 17.2	*0.048* *

Data are presented as Mean ± SD. The difference between control and laser treated eye was analysis by paired *t*-test.

* p < 0.05.

** p < 0.01.

*** p < 0.001.

## Data Availability

Data will be made available on request.

## References

[R1] Abbott CJ, Choe TE, Lusardi TA, Burgoyne CF, Wang L, Fortune B, 2014. Evaluation of retinal nerve fiber layer thickness and axonal transport 1 and 2 weeks after 8 hours of acute intraocular pressure elevation in rats. Investig. Ophthalmol. Vis. Sci 55, 674–687. 24398096 10.1167/iovs.13-12811 PMC3915863

[R2] Asrani SG, McGlumphy EJ, Al-Aswad LA, Chaya CJ, Lin S, Musch DC, Pitha I, Robin AL, Wirostko B, Johnson TV, 2024. The relationship between intraocular pressure and glaucoma: an evolving concept. Prog. Retin. Eye Res 103, 101303. 39303763 10.1016/j.preteyeres.2024.101303 PMC12556014

[R3] Chan KKW, Tang F, Tham CCY, Young AL, Cheung CY, 2017. Retinal vasculature in glaucoma: a review. BMJ Open Ophthalmol 1, e000032. 10.1136/bmjophth-2016-000032 PMC5721639 29354699

[R4] Chauhan BC, Pan J, Archibald ML, LeVatte TL, Kelly ME, Tremblay F, 2002. Effect of intraocular pressure on optic disc topography, electroretinography, and axonal loss in a chronic pressure-induced rat model of optic nerve damage. Investig. Ophthalmol. Vis. Sci 43, 2969–2976. 12202517

[R5] Dey A, Manthey AL, Chiu K, Do CW, 2018. Methods to induce chronic ocular hypertension: reliable rodent models as a platform for cell transplantation and other therapies. Cell Transplant. 27, 213–229. 29637819 10.1177/0963689717724793 PMC5898687

[R6] Feng KM, Tsung TH, Chen YH, Lu DW, 2023. The role of retinal ganglion cell structure and function in glaucoma. Cells 12. 10.3390/cells12242797 PMC10741833 38132117

[R7] Huang X-R, Zhou Y, Knighton RW, Kong W, Feuer WJ, 2012. Wavelength-dependent change of retinal nerve fiber layer reflectance in glaucomatous retinas. Investig. Ophthalmol. Vis. Sci 53, 5869–5876. 22836775 10.1167/iovs.12-10001 PMC3428115

[R8] Huang X, Kong W, Zhou Y, Gregori G, 2011. Distortion of axonal cytoskeleton: an early sign of glaucomatous damage. Investig. Ophthalmol. Vis. Sci 52, 2879–2888. 21245391 10.1167/iovs.10-5929 PMC3109006

[R9] Gallego BI, Salazar JJ, de Hoz R, Rojas B, Ramirez AI, Salinas-Navarro M, Ortin-Martinez A, Valiente-Soriano FJ, Aviles-Trigueros M, Villegas-Perez MP, Vidal-Sanz M, Trivino A, Ramirez JM, 2012. IOP induces upregulation of GFAP and MHC-II and microglia reactivity in mice retina contralateral to experimental glaucoma. J. Neuroinflammat 9, 92. 10.1186/1742-2094-9-92 PMC3410794 22583833

[R10] Huang XR, Knighton RW, Shestopalov V, 2006. Quantifying retinal nerve fiber layer thickness in whole-mounted retina. Exp. Eye Res 83, 1096–1101. 16828473 10.1016/j.exer.2006.05.020

[R11] Johnson TV, Tomarev SI, 2010. Rodent models of glaucoma. Brain Res. Bull 81, 349–358. 19379796 10.1016/j.brainresbull.2009.04.004 PMC2830899

[R12] LaVail MM, Battelle BA, 1975. Influence of eye pigmentation and light deprivation on inherited retinal dystrophy in the rat. Exp. Eye Res 21, 167–192. 1164921 10.1016/0014-4835(75)90080-9

[R13] Lin SC, Singh K, Jampel HD, Hodapp EA, Smith SD, Francis BA, Dueker DK, Fechtner RD, Samples JS, Schuman JS, Minckler DS, American Academy of, O., Ophthalmic Technology Assessment Committee Glaucoma, P., 2007. Optic nerve head and retinal nerve fiber layer analysis: a report by the American academy of ophthalmology. Ophthalmology 114, 1937–1949. 17908595 10.1016/j.ophtha.2007.07.005 PMC3780976

[R14] Liu X, Li M, Zhong Y-M, Xiao H, Huang J-J, Kong X-Y, 2010. Damage patterns of retinal nerve fiber layer in acute and chronic intraocular pressure elevation in primary angle closure glaucoma. Int. J. Ophthalmol 3, 152–157. 22553541 10.3980/j.issn.2222-3959.2010.02.14 PMC3340777

[R15] Lommatzsch C, van Oterendorp C, 2024. Current status and future perspectives of optic nerve imaging in glaucoma. J. Clin. Med 13. 10.3390/jcm13071966 PMC11012267 38610731

[R16] Morrison JC, Johnson E, Cepurna WO, 2008. Rat models for glaucoma research. In: Nucci C, Cerulli L, Osborne NN, Bagetta G (Eds.), Progress in Brain Research. Elsevier, pp. 285–301. 10.1016/S0079-6123(08)01121-7 18929117

[R17] Nadal-Nicolas FM, Vidal-Sanz M, Agudo-Barriuso M, 2018. The aging rat retina: from function to anatomy. Neurobiol Aging 61, 146–168. 29080498 10.1016/j.neurobiolaging.2017.09.021

[R18] Pitha I, Du L, Nguyen TD, Quigley H, 2024. IOP and glaucoma damage: the essential role of optic nerve head and retinal mechanosensors. Prog. Retin. Eye Res 99, 101232. 38110030 10.1016/j.preteyeres.2023.101232 PMC10960268

[R19] Prieto-Lopez L, Pereiro X, Vecino E, 2024. The mechanics of the retina: muller glia role on retinal extracellular matrix and modelling. Front. Med 11, 1393057. 10.3389/fmed.2024.1393057 PMC11410058 39296899

[R20] Quigley HA, Broman AT, 2006. The number of people with glaucoma worldwide in 2010 and 2020. Br. J. Ophthalmol 90, 262–267. 16488940 10.1136/bjo.2005.081224 PMC1856963

[R21] Quigley HA, Miller NR, George T, 1980. Clinical evaluation of nerve fiber layer atrophy as an indicator of glaucomatous optic nerve damage. Arch. Ophthalmol 98, 1564–1571. 7425916 10.1001/archopht.1980.01020040416003

[R22] Rodriguez AR, de Sevilla Muller LP, Brecha NC, 2014. The RNA binding protein RBPMS is a selective marker of ganglion cells in the mammalian retina. J. Comp. Neurol 522, 1411–1443. 24318667 10.1002/cne.23521 PMC3959221

[R23] Rovere G, Nadal-Nicolas FM, Agudo-Barriuso M, Sobrado-Calvo P, Nieto-Lopez L, Nucci C, Villegas-Perez MP, Vidal-Sanz M, 2015. Comparison of retinal nerve fiber layer thinning and retinal ganglion cell loss after optic nerve transection in adult albino rats. Investig. Ophthalmol. Vis. Sci 56, 4487–4498. 26193926 10.1167/iovs.15-17145

[R24] Sappington RM, Carlson BJ, Crish SD, Calkins DJ, 2010. The microbead occlusion model: a paradigm for induced ocular hypertension in rats and mice. Investig. Ophthalmol. Vis. Sci 51, 207–216. 19850836 10.1167/iovs.09-3947 PMC2869054

[R25] Sarthy V, Ripps H, 2001. The Retinal Müller Cell : Structure and Function. Kluwer Academic/Plenum Publishers, New York.

[R26] Schuman JS, Hee MR, Puliafito CA, Wong C, Pedut-Kloizman T, Lin CP, Hertzmark E, Izatt JA, Swanson EA, Fujimoto JG, 1995. Quantification of nerve fiber layer thickness in normal and glaucomatous eyes using optical coherence tomography. Arch. Ophthalmol 113, 586–596. 7748128 10.1001/archopht.1995.01100050054031

[R27] Song Y, Zhao L, Tao W, Laties AM, Luo Z, Wen R, 2003. Photoreceptor protection by cardiotrophin-1 in transgenic rats with the rhodopsin mutation s334ter. Investig. Ophthalmol. Vis. Sci 44, 4069–4075. 12939330 10.1167/iovs.02-1130

[R28] Tu S, Li K, Ding X, Hu D, Li K, Ge J, 2019. Relationship between intraocular pressure and retinal nerve fibre thickness loss in a monkey model of chronic ocular hypertension. Eye (Lond) 33, 1833–1841. 31227788 10.1038/s41433-019-0484-1 PMC7002733

[R29] Vidal-Sanz M, Salinas-Navarro M, Nadal-Nicolas FM, Alarcon-Martinez L, Valiente-Soriano FJ, de Imperial JM, Aviles-Trigueros M, Agudo-Barriuso M, Villegas-Perez MP, 2012. Understanding glaucomatous damage: anatomical and functional data from ocular hypertensive rodent retinas. Prog. Retin. Eye Res 31, 1–27. 21946033 10.1016/j.preteyeres.2011.08.001

[R30] Vrabec JP, Levin LA, 2007. The neurobiology of cell death in glaucoma. Eye (Lond) 21 (Suppl. 1), S11–S14. 18157171 10.1038/sj.eye.6702880

[R31] Wang H, Peng Z, Li Y, Sahn JJ, Hodges TR, Chou TH, Liu Q, Zhou X, Jiao S, Porciatti V, Liebl DJ, Martin SF, Wen R, 2022. sigma(2)R/TMEM97 in retinal ganglion cell degeneration. Sci. Rep 12, 20753. 36456686 10.1038/s41598-022-24537-3 PMC9715665

[R32] Wang L, Cioffi GA, Cull G, Dong J, Fortune B, 2002. Immunohistologic evidence for retinal glial cell changes in human glaucoma. Investig. Ophthalmol. Vis. Sci 43, 1088–1094. 11923250

[R33] Weinreb RN, Aung T, Medeiros FA, 2014. The pathophysiology and treatment of glaucoma: a review. JAMA 311, 1901–1911. 24825645 10.1001/jama.2014.3192 PMC4523637

[R34] Zhang J, Li L, Huang H, Fang F, Webber HC, Zhuang P, Liu L, Dalal R, Tang PH, Mahajan VB, Sun Y, Li S, Zhang M, Goldberg JL, Hu Y, 2019. Silicone oil-induced ocular hypertension and glaucomatous neurodegeneration in mouse. eLife 8. 10.7554/eLife.45881 PMC6533060 31090540

